# Biomimetic Nanocarriers for Cancer Target Therapy

**DOI:** 10.3390/bioengineering7030111

**Published:** 2020-09-14

**Authors:** Clara Guido, Gabriele Maiorano, Barbara Cortese, Stefania D’Amone, Ilaria Elena Palamà

**Affiliations:** 1 Department of Mathematics and Physics, University of Salento, Monteroni Street, 73100 Lecce, Italy; clara.guido@nanotec.cnr.it; 2 Nanotechnology Institute, CNR-NANOTEC, Monteroni Street, 73100 Lecce, Italy; gabriele.maiorano@nanotec.cnr.it (G.M.); stefania.damone@nanotec.cnr.it (S.D.); 3 Nanotechnology Institute, CNR-NANOTEC, c/o La Sapienza University, Piazzale A. Moro, 00185 Rome, Italy; barbara.cortese@nanotec.cnr.it

**Keywords:** biomimetic nanoparticles, cancer therapy, immunotherapy

## Abstract

Nanotechnology offers innovative tools for the design of biomimetic nanocarriers for targeted cancer therapy. These nano-systems present several advantages such as cargo’s protection and modulation of its release, inclusion of stimuli-responsive elements, and enhanced tumoral accumulation. All together, these nano-systems suffer low therapeutic efficacy in vivo because organisms can recognize and remove foreign nanomaterials. To overcome this important issue, different modifications on nanoparticle surfaces were exploited in order to reach the desired therapeutic efficacy eliciting, also, the response of immune system against cancer cells. For this reason, more recently, a new strategy involving cell membrane-covered nanoparticles for biomedical application has been attracting increasing attention. Membranes from red blood cells, platelets, leukocytes, tumor, and stem cells, have been exploited as biomimetic coatings of nanoparticles for evading clearance or stimulated immune system by maintaining in the same way their targeting capability. In this review, the use of different cell sources as coating of biomimetic nanocarriers for cancer therapy is discussed.

## 1. Introduction

Cancer remains one of the main reasons of death, second only to cardiovascular diseases [1,2]. Classical methods of treatment include surgery, radiotherapy, and chemotherapy. Surgery and radiation are still used, today, as localized treatments, affecting only small areas of the body. Chemotherapy has been mainly employed in the past because the early developed chemotherapeutic agents, interfering with DNA-coping process, affected all the replicating cells. From the discovery of the therapeutic effects exerted by nitrogen mustard and antifolates on cancer in the 1940s, many scientific progresses occurred in leading to the identification of distinctive targets and, consequently to newer pharmacological agents for specific treatment [3]. Despite the huge improvements in the beneficial effects with the development of chemotherapeutic drugs, setbacks are still related to their adverse toxic effects in different tissues and organs due to off-target accumulation and drug resistance. To overcome these issues, nanodrugs, which refer to the application of functionalized nano-carriers in pharmacology, represent a new opportunity for improved therapeutic efficiency, paving the way for more personalized cancer therapies [4,5]. The concept of attacking and destroying cell tumors by simply manipulating irregularities of tumor blood vessels to obtain an effective and selective drug accumulation has generated huge efforts in the scientific community toward the construction of nanocarriers for passive targeting, without the conjugation of specific ligands on NP surfaces’ [6,7]. Nanocarrier systems can be typically of different materials, such as lipids, polymers, polymer-drug conjugates, metals, in order to obtain organic and inorganic nanoparticles (NPs). Reports of these NPs have described prolonged circulation properties and an enhanced tumor accumulation over time due to the enhanced permeability and retention (EPR) effect [8] which originates from structural irregularity, heterogeneity, and leakiness of the tumor vessels along with lack of lymphatic drainage. However, in order to achieve a successful passive targeting by means of the EPR effect, NPs must fulfil several requirements, such as biocompatibility, long-circulation time and selectively. In fact, several physiological responses can harness the therapeutic efficacy of the nanodrugs: for example, immune cells can internalize the nanomaterials. This, however, raise concerns about their (specific) immunogenicity. In fact, recent evidences reported that some organisms have shown to can recognize and eliminate foreign elements, leading to a reduced NP uptake by the reticuloendothelial system (RES) especially in unvascularized cell clusters of the metastasis lesions [9,10]. In this frame, the physicochemical properties of nanomaterials are not the only challenge. In fact, NPs dispersed in biological solutions can adsorb proteins in heterogeneous networks, as what is denoted as the “corona” concept [11,12,13]. The protein corona can thus mediate the clearance of NPs by RES as well as the intrinsic, potential immunogenity of the nanomaterial [9]. For these reasons, nanocarriers have evolved toward complex chemical architectures in order to carry also specific chemical functionalities, able to preferentially target the site of interest with their payload while avoiding unwanted immune clearance. Several strategies have thus been employed to obtain the correct balance between active targeting and stealth properties to the immune surveillance of the host. The most common approaches strive to extend the blood circulation time and to limit the recognition by the immune system by grafting of Poly (ethylene glycol) (PEG) onto the NP’s surface. This layer aids to increase the NP uptake time in vivo from an uptake lasting minutes for uncoated NPs, till hours for NPs coated with PEG [14,15]. However, repeated administration of PEG-coated NPs, with specific antibodies IgM for PEG can lead to an accelerated clearance of the nanodrugs by the liver [16].

Hence, there is still need to pursue other strategies in order to produce actively targeted nanomedicines able to reach clinical trials. For this reason, more recently, an innovative approach involving cell membrane-coated (CMC) NPs has emerged, owing to their self-recognized property, immune elusion and long-time circulation in vivo. In particular, membranes deriving from red blood cells (RBC), platelets, leukocytes, cancer and stem cells are used to mask NPs in order to elude immune clearance [17,18]. Specifically, cell membranes devoid of cytoplasm and organelles denoted as “cell ghosts” [19] can express precise markers for suitable NP distribution, being structurally and functional like their source cells from which they derive. They also allow a direct coating of NPs avoiding additional chemical modifications. The resulting cell-ghost-coated NPs, thus, display a biologically intact bilayer membrane, while mimicking the surfaces of the source cells, with the potential to increase the biocompatibility of the nanocarriers, and achieving efficient and prolonged circulation in vivo, as well as performance of targeted purposes [17].

The use of cell membranes in biomimetic approaches confer a specific biological identity with well-defined molecular interactions owing to the presence of a structured arrays of membrane proteins. However, the experimental steps necessary for the production of these complex NPs may introduce new features in the membrane by altering membrane protein stability, composition, orientation, and glycosylation [20], leading to potential different biological interactions. For this reason, the interactions occurring between biomimetic NPs and biological components is an important issue to be addressed. However, to the best of our knowledge, few studies have explored the protein corona formation onto biomimetic NPs. Corbo and colleagues studied the protein corona formation in vivo of biomimetic leukocyte-like liposomes, named leukosomes [21]. They demonstrated that these complex nanostructures absorbed fewer proteins with respect to liposomes (without leukocyte membrane proteins inserted in the liposomal lipid bilayer) employed as control. This was ascribed to the qualitative differences in the protein composition between leukosomes and liposomes. In another study, NPs coated with RBC membranes showed, virtually, no protein adsorption when exposed to human plasma [22].

### Methods of Production of CMC-NPs

**Figure 1:**
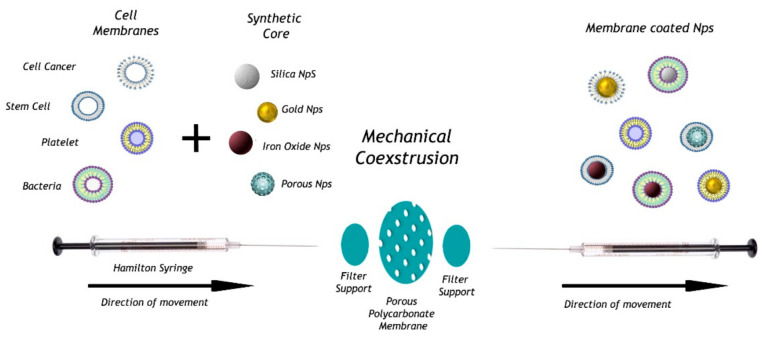
Schematization of co-extrusion method for cell membrane-coated nanoparticles (CMC-NPs).

Depending on the purpose and target disease, it is possible to produce CMC from different cell lines i.e., cancer and stem cells, platelets, RBCs, and leukocytes thus allowing a wide array of plasma membranes. Regardless the cell sources, the conventional method required for the production of CMC-NPs can be summarized in three crucial phases: membrane withdrawal, inner core nanocarrier making, and combination final procedure [17,18,19], as depicted in Figure 1.

The entire procedure is simple and does not require complex instrumentations. First the membrane, contingent the cell nature used, is extracted. For instance, RBCs and platelets, which are both without nuclei, can be directly isolated from whole blood sample [23], whereas, leukocytes and other cells must be separated from tissues or blood and then cultured in vitro [24,25]. Second, the inner core of the nanodrug must be obtained (using nanoprecipitation, double-emulsion or other methods). Several materials, mainly FDA approved polymers such as poly(lactic-co-glycolic) acid (PLGA), poly lactic acid (PLA), and poly caprolactone (PCL), but also inorganic polymers (black phosphorus and others) as well as metals like gold are usually exploited. Subsequently, in order to cover these nanocarriers with the membrane, a process of membrane extrusion or alternatively ultrasonic fusion leads to the self-assembly of the two components [13,14]. For cell membrane extrusion, a nanoscale polycarbonate porous membrane is used to obtain the combination of the CMC and inner core nanocarrier inside the NPs. An alternative approach to promote the access of NPs into cell membranes is the microfluidic electroporation [26]. This process can effectively aid to the synthesis of NPs, separating the dielectric layer of cell membranes, using electroporation, and creating several transient pores for the access of the NPs through the use of a microfluidic device. Moreover, advantages of this technique are the improved transfection performance, high throughput, and quantitative format. Once these new biomimetic NPs are obtained, prior studying their biological activities, several physicochemical characterizations must be carried out. Scanning and transmission electron microscope (SEM and TEM), dynamic light scattering (DLS) analyses allow to study and compare morphology, size, and surface charge; biochemical methodologies such as SDS-Page and Western blotting are required to check the presence of the specific membrane protein markers and therefore its functionality. These analyses are crucial for biomedical applications [7,13,14].

In this review, the different source cells as coatings of biomimetic nanocarriers for cancer therapy, also referring to the most recent clinical trials, will be discussed.

## 2. Cancer CMC NPs

The adhesion molecules present on the membrane’s surface of cancer cells play a crucial part in the process of cancer development and in metastasis formation, through cell-cell heterotypic (among cancer cells and other cell types) or homotypic (among similar cancer cells) adhesive contacts. It is now, well renown that cancer can interact through a homotypic aggregation which prevents the clearance of the metastatic cells. These cell-cell and cell-matrix interactions are mediated by specific molecules presents on their surface, such as integrins, selectins, E-cadherins, Thomsen-Friedenreich (TF) antigens, and the immunoglobulin superfamily (Ig-SF) [18,27,28]. Specifically, in the homotypic aggregation of circulating tumoral cells, the membrane proteins TF-antigen and E-cadherin seem to be involved [29]. In addition, special attention should be paid to CD47, overexpressed on tumoral cells and reliable for the immune-escaping assets of cancer which depends on the interaction with signal regulatory protein-α (SIRP-α) manifested by macrophages and dendritic cells. The linking of SIRP-α with CD47 leads to phosphorylation of the cytoplasmic extremity of SIRP-α, followed by activation of protein phosphatases and blockage of the phagocytosis [30]. In this way, this protein prevents the macrophages uptake of cancer cells. The advantages using cancer-CMC-NPs are immune-evading properties (CD47), homotypic targeting behavior (cadherin, integrins), and cancer immunotherapy (vaccination) with improved tumor-specific accumulation, enhanced circulation, and enhanced drug or gene delivery avoiding side effects. CMC has also shown inhibition of early release of therapeutic agent in the blood [31].

The homotypic targeting behavior of CMC-NPs represents a key factor in delivering therapeutic active agents to the specific site. Fang’s workgroup [19] synthesized PLGA NPs with plasma membranes from a MDA-MB-435 tumoral cell line, reporting homotypic targeting for MDA-MB-435 tumor and specific drug delivery. Core-shell PLGA NPs coated with cell surface adhesion domains present on MDA-MB-435 cells, were reported with a robust homotypic attraction with the tumor cells of origin, leading to an improved cellular uptake with respect to bare PLGA-NPs. In addition, Fang’s group showed that these NPs can stimulate the maturation of T-cells if administrated together with an adjuvant. Briefly, these biomimetic cancer cell NPs were loaded with monophosphoryl lipid A (MPLA) that was utilized as adjuvant molecule, and incubation with mice dendritic cells resulted in a significant immune response derived by upregulation of the dendritic maturation markers CD40, CD80, and CD86. In addition, quantification of the interferon-gamma (IFNγ) demonstrated that CMC-NPs loaded with MPLA were capable to provoke an antigen response. So, the dual main goal that can be achieved using cancer CMC-NPs, can be summarized as “homotypic targeting” to increase targeted drug delivery, and improved immunotherapy due to the antigen delivery.

**Figure 2:**
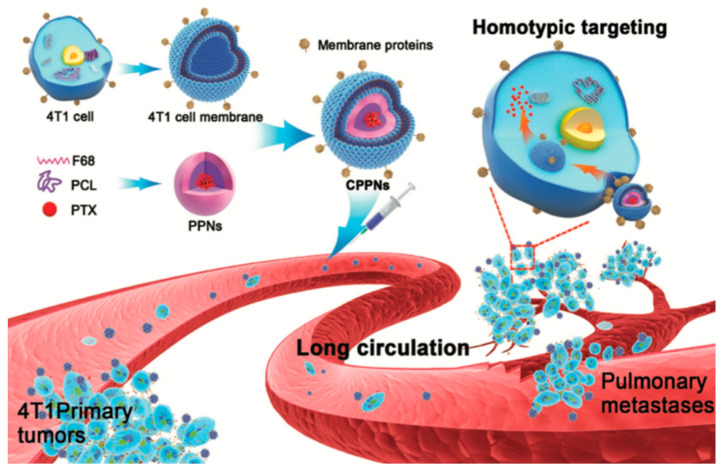
Schematization of homotypic cancer targeting using CMC-NPs. Reproduced, with permission, from Ref. [32].

Sun et al. [32] formulated polymeric NPs composed by PCL and pluronic copolymer F68, loaded with paclitaxel and covered with CMC obtained by 4T1 breast cancer cells. His group confirmed that breast tumor cells, both from the metastasis lesion and from the primary tumor, were recognized by the biomimetic NPs owing to the presence of the surface proteins essential for the homotypic binding. Comparing membrane-coated NPs to uncoated NPs, they observed an enhanced uptake in presence of membrane, and the quantitative results revealed that membrane-coated NPs accumulated into primary tumors and in metastases more effectively than uncoated NPs (Figure 2). The homotypic targeting was effectively confirmed by lower cellular uptake obtained with the same NPs coated with different membranes derived from other type of cells. Similar results were also evident using as a negative control fibroblast WML2. Also, the immune evading properties were confirmed, owing to the surface protein CD47 which showed to increase the cellular uptake of membrane-coated NPs when compared to naked.

Application of these nanocarriers as cancer nanovaccines has also been reported [24]. PLGA NPs with an agonist of Toll-like receptor 7 as cargo, and coated with melanoma cell membrane modified with mannose, promoted the interaction with DCs. Exploiting proteins of cancer cell membranes, which can act as tumor specific antigens, has shown to improve uptake by DCs, followed by the stimulation into the maturation status which led to an enhanced anti-tumor immune response. In the final outcomes, these biomimetic NPs, embodied both vaccines protecting mice from cancer cells and fighting melanoma progression.

Therefore, biomimetic cancer cell membrane-coated NPs can express tumoral-associated antigens (TAAs) on their surface and stimulate dendritic cells eliciting the immune responses against the TAAs.

## 3. Leucocyte Cell Membrane-Covered NPs

Leukocytes or white blood cells (WBCs), are cells of immune system indispensable for defense of the host against pathogen attack and diseases [33]. These cells are, in dimension, considerably bigger than RBCs. They can display a faster and efficient extravasation from the blood into the neighboring tissues, leading them to be profuse both in the blood flow and in extravascular sites [34]. Importantly, they can interrelate with tumor cells either directly in the cancer environment or in blood flow owing to their adhesive properties. In order to carry out effectively the active therapeutic agents, the active therapeutic molecules must elude the phagocytic uptake, and target the desired site, circumventing any vascular walls to reach the targeted tissue. To surmount these tasks, leucocytes membrane-covered NPs seem to show promising applications.

Tasciotti’s group has demonstrated that membranes extracted by leukocytes used to coat porous silicon NPs were capable to bind the inflamed endothelial cells and locate to the specific cancer target [35]. The same group in another work [36] demonstrated that shelling NPs with leukocytes membrane, activated proficiently the signal pathway of the endothelial receptor ICAM-1, ensuing improved vascular penetrability owing to the VE-cadherin phosphorylation. Moreover, in vivo analysis showed that this method improved the targeting assets, endorsed adhesion to the cancer blood endothelium improving cancer perfusion. Zhang and co-workers [37] showed that macrophage membrane-covered NPs can be used for the controlled release of therapeutic agents in response to the pH of the cancer environment. After the outbreak of covered macrophage membranes by interstitial pH of cancer tissue, the innermost nanocores loaded with drug molecules were engulfed by cancer cells with release of drugs displaying good cancer-homing capacity in blood circulation.

In a recent study, J774 cell membranes were used to obtain WBC membrane-covered NPs. These WBC-coated NPs were loaded with doxorubicin (DOX) and showed an uptake of about 75% by J774 cells. These NPs were also capable to tie precisely to inflamed sites, facilitating transport of drug across the vasculature, guarantying the effective DOX delivery to the tumor site [35].

Cao and colleagues [38] reported membranes extracted by RAW264.7 macrophages and used to coat pH-sensitive liposomes loaded with chemotherapic emtansine, showing an enhanced drug delivery to metastases. Efficiency of uptake by 4T1 breast cancer cells was higher for the macrophage-covered NPs than for uncoated. Moreover, 4T1 lung metastases in vivo were inhibited by the biomimetic WBC-coated NPs of about 87%.

Also monocyte membranes revealed to be useful in biomimetic NPs for cancer therapy by exploiting cell adhesion molecules such as α4β1 of the circulating monocyte to produce CMC-NPs able to target breast cancer that overexpress cell adhesion molecules such as VCAM-1 [39,40].

Recently, T-cell-derived membranes have been used to produce biomimetic NPs owing to an extended blood circulation period and their aptitude to confine at cancer [35]. Also, they expressed higher levels of adhesion molecules than their naive equivalent cells and were shown to be efficient in targeting the cancer sites. An application of T cell membrane-coated NPs has been described by Zhang and coworkers [41]. This group produced PLGA NPs, loaded with paclitaxel and coated with the membrane of human cytotoxic CD8+ T lymphocytes (hCTLs). When used in combination with local low-dose irradiation (LDI), these biomimetic NPs, were capable to restrain cancer progression in a model of human gastric tumor of about 88%.

**Figure 3:**
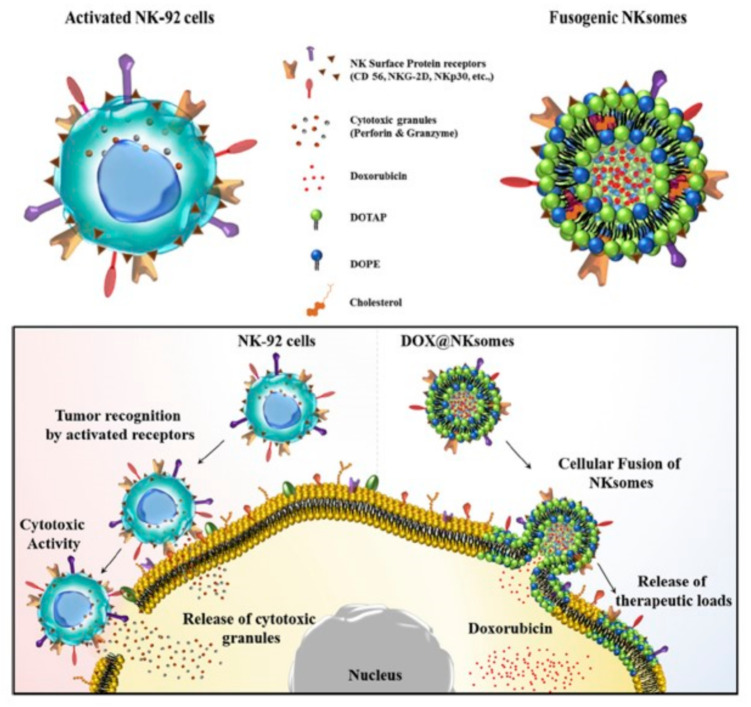
Illustration of target cancer therapy using activated natural killer (NK) cells and liposomes coated with NK membrane (NKsomes). Reproduced with permission from Ref. [42].

Moreover, the subset of lymphocytes named as natural killer (NK) cells have been recently employed to produce DOX-loaded liposomes coated with membranes derived by active NK [42], thus achieving efficient cancer targeting (Figure 3). The resulting “NKsomes”, revealed a higher and improved attraction for cancer cells in vitro and in vivo, with a longer circulation half-life. in vivo biodistribution and pharmacokinetic analysis showed tumor homing.

In another study [43], cell membrane coating of inflammatory neutrophils were used to prevent early metastasis, inhibiting the progression of already formed metastasis. PLGA NPs loaded with proteasome inhibitor carfilzomib, were shelled with neutrophil membranes. The resulting biomimetic NPs were capable to bind to circulating tumor cells (CTCs) leading to their apoptosis and targeting also the already formed metastatic 4T1 models through the interaction with the inflamed endothelial cells in the premetastatic lesion.

Thus, WBC-covered NPs act as an attractive method to evade NP immune recognition and help constant circulation as well as active therapeutic agents release.

## 4. Platelet Cell Membrane Covered NPs

Platelets derived from megakaryocyte progenitors can be produced in large quantities [44]. Platelet membranes have attracted considerable interest owing to their availability and exceptional physiological roles. Their ability to address vascular injury and to interact with circulating cancer cells, makes them as exceptional platforms for cancer targeting. Furthermore, platelet-biomimetic NPs exhibit higher blood circulation periods and a reduced engagement from healthy tissues. Specifically, platelet membranes offer potential advantages for NP coating, because of the existence of specific ligands on their surface such as CD47 that allow the immune elusion and CD55/59 that can avoid complement activation [45]. Furthermore, the presence of CD44 and P-selectin receptors on their surface allows binding to circulating cancer cells [46].

Recently, platelet membrane-coated nanovesicles (PMNVs) were produced co-loading DOX and ligand TRAIL able to induce apoptosis into target cells [46]. Their ability to delivery TRAIL to the membranes of MDA-MB-231 cells, provoking apoptosis has been clearly shown.

Moreover, platelet membrane-coated NPs loaded with DOX and modified with RGD peptides, were shown to be capable of avoiding immune-mediated purging and target cancer vasculature [47]. The unique properties of platelet membrane were employed also by Kim and co-workers to produce platelet membrane-coated gold nanostars containing curcumin with the ability to efficiently target melanoma cancer cells [48]. The platelet membrane was able to confer direct targeting capability to gold NPs as photothermal therapy with the anticancer and anti-inflammatory properties of the curcumin, while avoiding macrophage phagocytosis.

**Figure 4:**
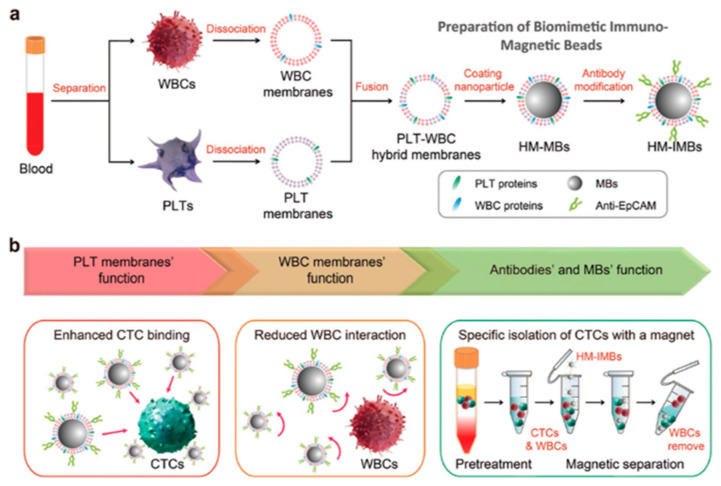
Schematic illustration of membrane-coated immunomagnetic beads (HM-IMBs). (**a**) Membrane isolated by WBC and PLT were utilized to coat the magnetic beads (MBs). (**b**) Membrane-coated immunomagnetic beads improved the binding of circulating tumoral cells (CTSs) from PLTs and abridged the homologous interaction with WBCs. Reproduced with permission from Ref. [23].

Another recent application of platelet membrane as NP coating was also addressed to target isolated circulating tumoral cells (CTSs) [18]. Briefly, magnetic beads were coated with platelet (PLT) and WBC membranes and their surface were modified with antibodies targeting CTCs. In this way, these PLT–WBC hybrid membrane-covered immunomagnetic beads (HM-IMBs) were used for the specific isolation of CTCs owing to the cancer cell binding ability of PLTs [49]. Thus, PLT-CTC interaction can improve the isolation of cancer cells, increasing at the same time their purity due to the reduced interaction with homologous WCs [50] and suppressing unspecific binding of leukocytes onto CTCs (Figure 4). When tested on human breast cancer MCF-7 cells these NPs revealed a higher capture efficiency after anti-EpCAM modification of their surface.

The unique features of platelet membranes offer important opportunities in the design of cell-based hybrid systems with the dual ability to target cancer and evade immune reactions, thus representing an area of interest that, however, needs further research.

## 5. Stem Cell Membrane-Coated NPs

Stem cells are renowned to self-renew and enable growth of several cell lines. Mesenchymal stem cells (MSCs) are adult stem cells which can be isolated from many tissues such as adipose tissue, peripheral blood, umbilical cord and placenta. These cells show unique biological properties and an extraordinary ability of in vitro growth which allows them to grow quickly, reaching the required quantity for in vivo treatment [51,52]. Moreover, mesenchymal stem cells are ideal for biomimetic NPs delivery due to many advantages such as extended circulation in blood, immune elusion, and cancer targeting features [25]. These latter characteristics depend on the expression of ligands fine-suited for cancer targeting, allowing them to travel to damaged tissues in vivo [53]. Several studies have reported that transplanted MSCs can selectively target a wide range of pathological tissues producing encouraging outcomes in numerous clinical trials with dissimilar illnesses such as solid and hematological tumors, degenerative diseases, and immune syndromes [54,55]. Several clinical trials have been performed, exploiting the MSC homing and tumor targeting ability [25].

**Figure 5:**
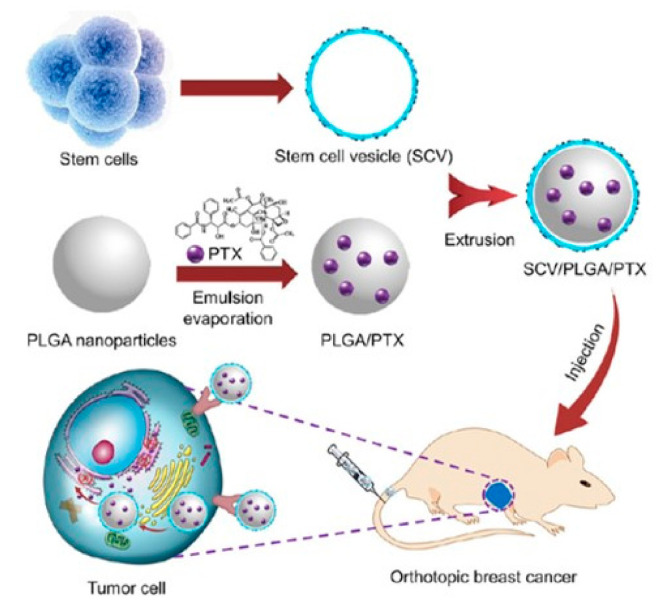
Schematization of PLGA NPs loaded with paclitaxel (PTX) covered with stem cell membranes and their mechanism of tumor target delivery in orthotopic breast cancer in mouse. Reproduced with permission from Ref. [56].

The group of Tian [56] showed how PLGA NPs loaded with paclitaxel (PTX) and coated with mesenchymal stem cell membranes can be effective in tumor chemotherapy in a orthotopic breast cancer in mouse, increasing time of circulation of NPs and the uptake, and displaying a controlled release of the payload in an efficient manner while avoiding important side effects (Figure 5).

In another recent study [57], PLGA NPs loaded with DOX were covered with umbilical cord MSC cells and tested on MHCC97-H liver tumor, exhibiting an inhibition tumor growth rate of about 78% in vivo. Same encouraging results were obtained also in another recent work [58], which reported biomimetic gelatin-based nanogels loaded with DOX, coated with MSC membrane. These NPs showed to be able to inhibit the growth of HeLa cells in comparison to free DOX and naked gelatin-DOX NPs, with improved therapeutic efficiency related to MSC membrane coating.

Kaneti et al. [59] produced NPs loaded with plasmid encoding for hemopexin-like domain (PEX), and coated with mesenchymal stem cells. These NPs were tested in two xenograft cancer models orthotopic metastatic pulmonary non-small cell lung carcinoma and subcutaneous prostate tumor showing specific apoptosis of cancer cells.

Membranes extracted using mesenchymal stem cells to coat NPs, owing to their many targeting moieties and their homing capability, result in a potential approach for tumor target treatment.

## 6. RBC Membrane-Covered NPs

RBC represent the prevalent cells in the human blood with a diameter of about 7 μm and do not present a nucleus. RBCs are capable to change in shape while travelling through the body and are simply isolated from the blood. Thus RBCs embody a possibly ideal source of cell membranes well appropriate for in vivo circulation through the blood vessels of patients [60].

A crucial point regards the presence of CD47 protein on their surface, which is a self-antigen and can lead to long-standing RBCs circulation in vivo (~120 days in human and ~50 days in mice) [61]. Therefore, RBCs can be used as source cell of membrane to deliver specific therapeutics agents by coating specific NPs. This is possible because RBCs are wholly biodegradable and non-toxic. Moreover, as the membrane of RBCs is semi-permeable, this allows constant drug release utilizing RBC membrane covered NPs. Because of these properties, RBCs can be used for the transport of several therapeutic active molecules, as well as proteins, nucleic acids, and drugs [62,63,64].

In recent studies [65,66], different type of NPs coated with RBC-membranes showed a long-circulation time and a controlled drug-release of the encapsulated drugs, such as DOX, with an increased LC_50_ when injected into mice. The application as long-circulation nanocarriers for drug delivery, has been also confirmed by another study [67] in which mesoporous silica NPs covered with an RBC membrane exhibited long-term circulation and allowed efficient DOX release. These NPs showed a relevant increase in the circulation time, because of the RBC membrane which allows immune evasion, along with a controlled release of DOX from NPs.

Another advantage concerns the possibility of RBC membrane-encapsulated NPs to be capable to overcome poor water solubility and important adverse effects of chemotherapeutic drugs [68]. For example, gambogic acid (GA) is a new anticancer complex that exhibits poor water solubility and high degree of adverse effects, with a limited clinical value. In a recent study [69], PLGA NPs covered with RBC membranes and loaded with GA, obtained in vitro anticancer efficiency and showed to able to inhibit subcutaneous cancer evolution in vivo. However, an equal quantity of naked GA was only slightly capable to control cancer growth and showed to be far less effective in vivo than in vitro.

Recently, RBC membrane-covered solid lipid NPs were synthetized and functionalized with T7 and NGR peptides absorbed on their surface [70]. Loading these NPs with vinca alkaloid vincristine (VCR), showed anti-glioma effectiveness both in vitro and in vivo. This strategy allowed these NPs to improve drug transport to the brain, overcoming the challenges represented by blood-brain-barrier (BBB) and blood-brain-tumor-barrier (BBTB).

In a similar way, RBC-NPs were modified by addition or recombinant anti-EGFR-iRGD to the NPs surface. This allowed these NPs to reach effective and exact cancer-targeting in a high EGFR-expressing colorectal cancer model, while NPs lacking peptide modification were less effective [71].

**Figure 6:**
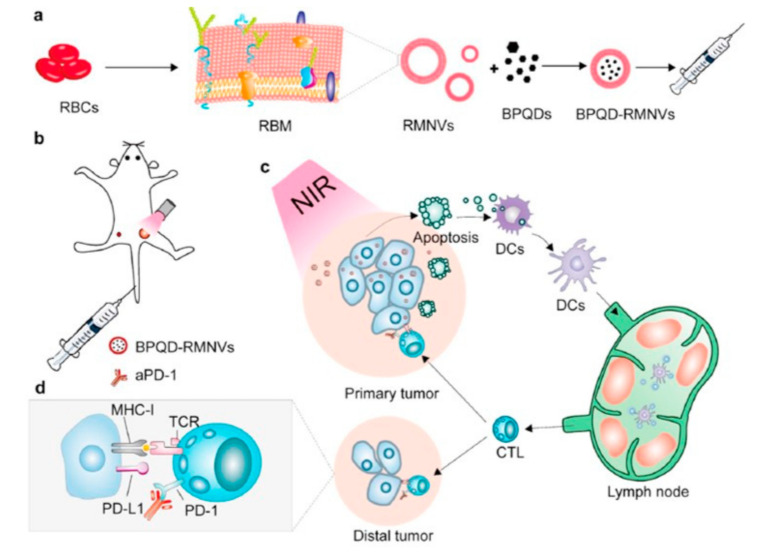
Schematic drawing of preparation of black phosphorous quantum dot (BPQDs) coated with RBC membrane (**a**); treatment of 4T1 tumor bearing mouse using BPQD coated with RBC membranes, aPD-1 and NIR (**b**); apoptosis of cancer cell and release of tumor antigens, dendritic cells (DC) recruitment for the exhibition of antigens to T-cells (**c**); aPD-1 working to keep tumor-infiltrating CD8+ T cells (**d**). Reproduced with permission from Ref. [72].

The use of biomimetic black phosphorous quantum dot (BPQDs) coated with RBC membrane has also been reported in combination with NIR irradiation and administration of PD-1 antibody leading to primary and secondary tumor inhibition [72] as schematized in Figure 6. Therefore, the combination therapy consisting of biomimetic NPs coated with RBC membranes, NIR and PD-1 antibody meaningfully deferred residual and metastatic cancer growth in mouse.

RBC cells represent a promising strategy to target therapeutic agent specific using biomimetic NPs with important application in cancer therapy due to their long time of circulation in vivo, to the presence of CD47 which allows to avoid phagocytosis from the immune system, for their semi-permeable membrane that allows a controlled sustained release.

## 7. Toxicity and Biological Impact of Biomimetic NPs

The toxicity and the biological impact of these biomimetic NPs produced from different source cells are an important issue to be addressed, although membrane-coated NPs allow to overcome problems associated with the administration of free therapeutic molecules, such as poor solubility on aqueous environment, non-specific target for cancer cells, and consequently side effects on healthy cells. Zhang et al. [69] tested PLGA NPs loaded with poor aqueous soluble gambogic acid and covered with RBC membranes on colorectal cancer cells. Along with uptake and anticancer activities studies, the biocompatibility of these NPs, with or without the cell membrane coating, was tested by incubation with macrophages. They showed a reduced uptake because of the presence of specific proteins on the RBC membrane, which allowed them to avoid phagocytosis, and increasing circulation time. In another similar recent study [41], biocompatibility assays over PLGA NPs carrying PTX drug, covered with cytotoxic T-lymphocytes membranes for the treatment of gastric cancer showed reduced take by macrophages with respect to the same NPs without hCTL membrane coverage. This result confirmed the property of these biomimetic structures to avoid the immune system. Moreover, cytotoxicity analysis showed similar decrease in cell viability over time, with increase of the drug concentration. In a recent study [73], synthesized PLGA NPs loaded with antitumor molecule bufalin, and covered with platelet membrane showed to avoid macrophages uptake and to increase the binding with target cancer cells. These biomimetic NPs showed to inhibit more effectively the cellular viability of H22 hepatoma cells with respect to free drug. Moreover, cellular uptake was higher by using NPs covered with platelet membrane, compared with uncoated. The hemolysis assay was then performed to assess their hemocompatibility, and the results showed only a percentage of 3.85% of RBC lysis, thus confirming blood compatibility. In order to study the biosafety in vivo, these biomimetic NPs were administrated in a mouse model of H22 tumor and no toxicity was observed when compared with the control group.

Sun et al. [74] developed PLGA NPs covered with macrophage membrane and loaded them with the anticancer drug Saikosaponin D to treat breast cancer and to overcome the drug toxicity. Because of the presence of T7 peptide on macrophages membrane, these NPs targeted breast cancer cells, showing an overexpression of the transferrin receptors. The uptake studies in cancer and healthy cells showed a higher uptake by cancer cells with respect to healthy cells. In addition, the cytotoxicity analysis showed a higher toxicity for cancer cells and no toxicity for healthy cells. Furthermore, the role of biomimetic NPs to the reduction of systemic toxicity in drug delivery has been reported also by Corbo and co-workers [21] together with the promising biocompatibility and biosafety profiles reported by Evangelopoulos and colleagues that demonstrated a minimal accumulation of biomimetic NPs in liver, lung, and spleen [75].

Despite the promising results of the mentioned works, studies on the bio-compatibility of NPs remain an important issue to be deeply explored for each biomimetic NP formulation. This is because these nano-bio hybrid NPs are designed to achieve prolonged circulation time and to avoid RES filtering, thus being more prone to elicit potential adverse effects. In order to overcome this potential limitation and to speed up their employment in clinical trials, the experimental steps to produce biomimetic NPs need to be carefully standardized among different laboratories in order to obtain reproducible nanostructures. As mentioned in the previous sections, the experimental steps necessary for the production of biomimetic NPs could alter the bio-chemical properties of the employed membrane through modification of membrane proteins stability, composition, orientation, and glycosylation thus representing a potential risk of unexpected immune response and adverse side effects. In fact, reports showed increase of toxicity for biomimetic NPs in correlation with conformational changes of the adsorbed proteins on the NPs’ surface [76]. Additionally, new strategies of functionalization are emerging in order to broaden the application of these membrane-covered NPs. These novel approaches are mainly based on lipid insertion, membrane hybridization, metabolic engineering, and genetic modification, as recently summarized in the review of Ai and colleagues [77], however standardization among approaches and laboratories is mandatory.

## 8. Conclusions

The field of drug and gene delivery has been widely studied for cancer therapy and several illnesses, leading researchers to look for a method that can satisfy the requirements of compatibleness for human use, and maximum efficiency in terms of care, so as to minimize treatment with free administered drugs or with classically used therapies that often lead to side effects. An ideal carrier should be biocompatible, biodegradable, and extremely safe, showing characteristics in relation with size, surface charge, and in general of membrane, which allows it to interact with the specific target, avoiding being recognized as “not self” by the immune system. To overcome these problems, recent research studies have thought to blend natural features of cell membranes with synthetic characteristics of NPs to design new carrier systems named as biomimetic NPs. Among the several sources of cells chosen, here we have discussed about RBCs, platelet, stem and tumor cells, and leucocytes, finding different advantages and characteristics in each of these species. It is clear that, as novel experimental strategies are developed to produce membrane-coated NPs, it is mandatory to standardize the production processes of the bio-chemical properties of the membrane through alteration of the surface and the biosafety assessing methods. Change of membrane proteins stability, composition, orientation, and glycosylation increase the probabilities of unexpected immune response and adverse side effects. This new field of nanotechnology paves the possibility to treat in an alternative manner important widespread diseases.
